# Electroencephalography Monitoring for Preventing Postoperative Delirium and Postoperative Cognitive Decline in Patients Undergoing Cardiothoracic Surgery: A Meta-Analysis

**DOI:** 10.31083/j.rcm2504126

**Published:** 2024-03-29

**Authors:** Song Xue, Ao-xue Xu, Hong Liu, Ye Zhang

**Affiliations:** ^1^Department of Anesthesiology and Perioperative Medicine, The Second Hospital of Anhui Medical University, 230061 Hefei, Anhui, China; ^2^Key Laboratory of Anesthesiology and Perioperative Medicine of Anhui Higher Education Institutes, Anhui Medical University, 230038 Hefei, Anhui, China; ^3^Department of Anesthesiology and Pain Medicine, University of California Davis Health, Sacramento, CA 95817, USA

**Keywords:** electroencephalography monitoring, postoperative delirium, postoperative cognitive decline, cardiothoracic surgery, cognitive dysfunction, postoperative cognitive complications, cognition disorders, delirium

## Abstract

**Background:**

Patients undergoing cardiothoracic surgery frequently encounter perioperative neurocognitive disorders (PND), which can include postoperative delirium (POD) and postoperative cognitive decline (POCD). Currently, there is not enough evidence to support the use of electroencephalograms (EEGs) in preventing POD and POCD among cardiothoracic surgery patients. This meta-analysis examined the importance of EEG monitoring in POD and POCD.

**Methods:**

Cochrane Library, PubMed, and EMBASE databases were searched to obtain the relevant literature. This analysis identified trials based on the inclusion and exclusion criteria. The Cochrane tool was used to evaluate the methodological quality of the included studies. Review Manager software (version 5.3) was applied to analyze the data.

**Results:**

Four randomized controlled trials (RCTs) were included in this meta-analysis, with 1096 participants. Our results found no correlation between EEG monitoring and lower POD risk (relative risk (RR): 0.81; 95% CI: 0.55–1.18; *p* = 0.270). There was also no statistically significant difference between the EEG group and the control group in the red cell transfusions (RR: 0.86; 95% CI: 0.51–1.46; *p* = 0.590), intensive care unit (ICU) stay (mean deviation (MD): –0.46; 95% CI: –1.53–0.62; *p* = 0.410), hospital stay (MD: –0.27; 95% CI: –2.00–1.47; *p* = 0.760), and mortality (RR: 0.33; 95% CI: 0.03–3.59; *p* = 0.360). Only one trial reported an incidence of POCD, meaning we did not conduct data analysis on POCD risk.

**Conclusions:**

This meta-analysis did not find evidence supporting EEG monitoring as a potential method to reduce POD incidence in cardiothoracic surgery patients. In the future, more high-quality RCTs with larger sample sizes are needed to validate the relationship between EEG monitoring and POD/POCD further.

## 1. Introduction

The number of older adult patients undergoing surgery is increasing as the population age increases and surgical techniques improve, which has led to an increased interest among anesthesiologists and surgeons in perioperative neurocognitive disorders (PND). PND is a common complication that occurs after major surgeries, including postoperative delirium (POD), postoperative cognitive decline (POCD), and delayed neurocognitive recovery [1]. Evidence has highlighted that the incidence of POD ranges from 11% to 51% across different surgical procedures [2]. POCD has been reported to occur in 25%–40% of cases [3]. POD and POCD are associated with prolonged hospital stays, increased hospitalization costs, higher mortality rates, and delayed recovery [4, 5].

POD refers to a sudden state of confusion in consciousness, perception, memory, and orientation, which often occurs soon after surgery [6]. Alternatively, POCD is characterized by difficulties in memory, perceptual motor function, learning, communication, and more, which typically occur later in the postoperative period [6]. A recent study by Glumac *et al*. [7] found that POD can predict postoperative cognitive dysfunction, which has significant consequences for patient health and the healthcare system. POD and POCD have different pathogenesis, although they share similar risk factors [8]. Previous studies have demonstrated that long-term exposure to anesthesia could cause neurotoxicity, leading to POCD and POD [9, 10]. In recent years, intraoperative brain function monitoring has become increasingly common in various surgical procedures. Electroencephalography (EEG) is frequently utilized to control the level of anesthesia and adjust the amount of anesthesia medication. The occurrence of POD and POCD has been observed to decrease with the use of EEG monitoring [11, 12]. However, certain clinical trial findings contradict this perspective [13, 14]. The use of EEG in preventing POD and POCD among older adult patients still lacks convincing evidence.

The occurrence of POD and POCD could differ based on the types of surgical procedures [5]. It has been suggested that the incidence of POD and POCD is relatively high in cardiac surgery [15, 16]. It was also confirmed that cardiothoracic surgery is a risk factor that contributes to PND [17]. However, previous meta-analyses have primarily focused on the relationship between EEG and anesthesia for non-cardiothoracic surgery [18, 19]. Fewer clinical studies focus on the impact of EEG on POD and POCD in patients undergoing cardiothoracic surgery.

Due to the uncertainty regarding the impact of EEG on POD and POCD in patients with cardiothoracic surgery, we conducted this meta-analysis to evaluate the relevance between EEG monitoring and POD/POCD in cardiothoracic surgery patients.

## 2. Materials and Methods

In this meta-analysis, we discussed the correlation between EEG and POD/POCD in patients who have undergone cardiothoracic surgery. Our study included several randomized controlled trials (RCTs). We followed the Preferred Reporting Items for Systematic Reviews and Meta-Analysis Statement (PRISMA) guidelines to conduct this meta-analysis [20]. This meta-analysis has been registered in the PROSPERO International Prospective Register of Systematic Reviews (CRD42023452498).

### 2.1 Search Strategy

Two investigators (SX and AXX) thoroughly searched the Cochrane Library, PubMed, and EMBASE databases independently from inception to August 15, 2023. We imposed no language or other limitations when conducting the literature search. The search keywords included “electroencephalography”, cardiothoracic surgery”, “postoperative delirium”, “postoperative cognitive decline”, and “randomized controlled trial”. We searched only human studies, meaning animal studies were excluded from the search. The two investigators dealt with any disagreements regarding search results and resolved them following a discussion.

### 2.2 Eligibility Criteria

We carefully reviewed literature that met the specified inclusion and exclusion criteria. The criteria for inclusion were as follows: (1) Study design: RCT; (2) participants: Adult patients undergoing cardiothoracic surgery; (3) intervention: EEG vs. routine monitor; (4) postoperative outcomes: POD and/or POCD. The exclusion criteria were: (1) non-RCT, (2) duplicates, (3) protocols or ongoing research, and (4) the outcome data were unavailable.

### 2.3 Data Extraction 

Data from the included study were extracted independently by two investigators (SX and AXX). Any disagreements regarding extractable information were resolved through discussions. The extracted information comprised the first author’s name, publication year, country, number and age of patients, intervention groups, POD/POCD assessment method, and follow-up period.

### 2.4 Quality Assessment

Two authors independently assessed the methodological quality of the included studies using the Cochrane tool (version 2, Cochrane, London, UK). A consensus meeting was held to resolve any disagreements. We assessed bias risk in various aspects and categorized it as low risk of bias, high risk of bias, and unclear. The quality assessment results were displayed through a risk-of-bias graph and summary Figure using Review Manager software (version 5.3, Cochrane, London, UK).

### 2.5 Statistical Analysis

We used Review Manager (version 5.3) to analyze the data from the included literature. For dichotomous variables, the risk ratio (RR) with a 95% confidence interval (CI) was used to determine the effect. RR was calculated using the Mantel–Haenszel (M–H) method. For continuous variables, we estimated the effect using mean difference (MD) and 95% CI. Inverse variance models were used to analyze MD. We used the chi-square test and *$I^{2}$* test to assess the heterogeneity between various trials. The random effects model was adopted if *$I^{2}$* >50% or *p* < 0.10, indicating high heterogeneity. Otherwise, a fixed effect model was used. To assess the impact of each trial on the overall results, we performed a sensitivity analysis, which involved deleting each study and then merging the RR values of the remaining trials.

## 3. Results

### 3.1 Literature Retrieval

**Fig. 1.**
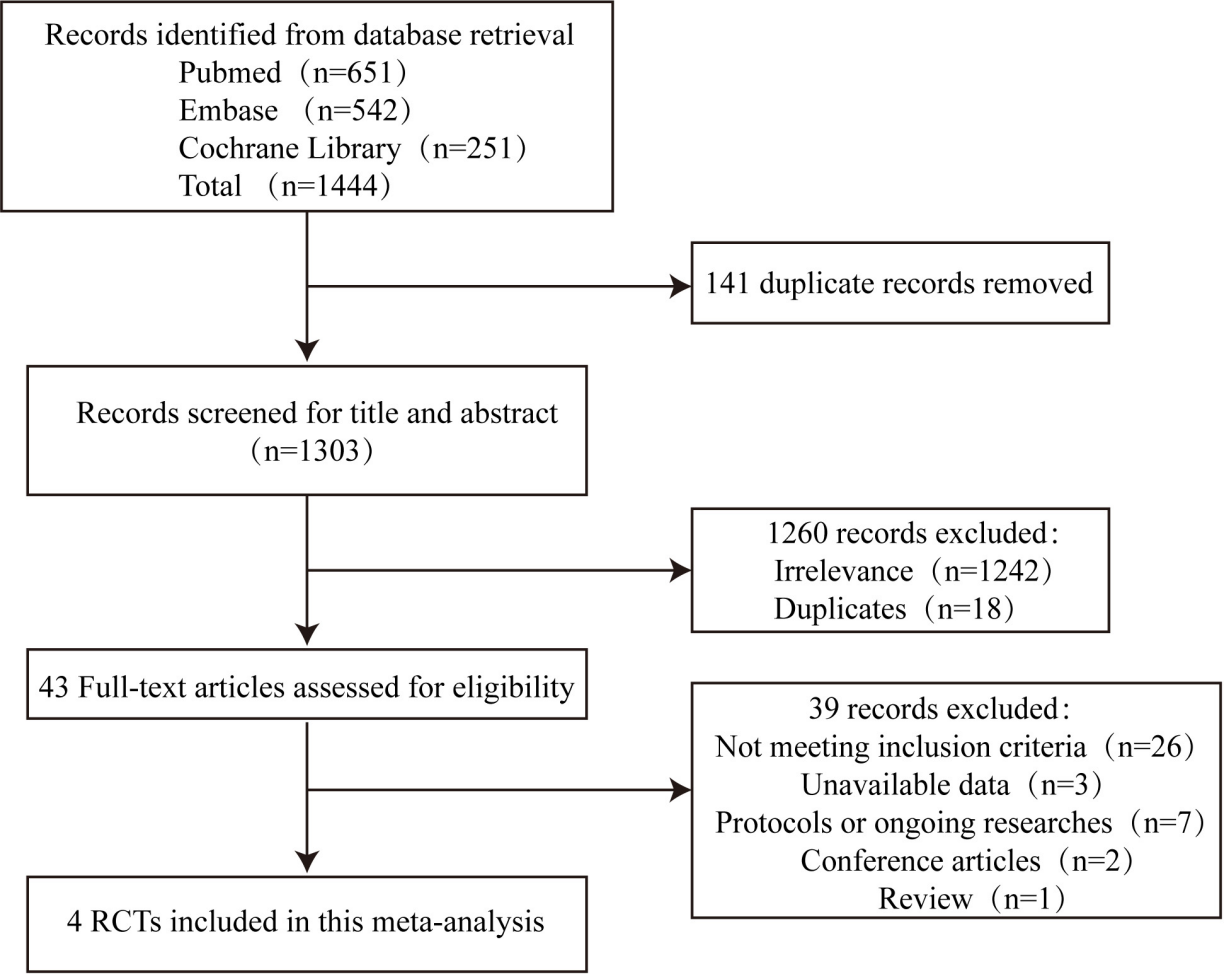
**Flow diagram of the literature search**. RCTs, randomized controlled trials.

Following a thorough database search, we acquired a preliminary collection of 1444 articles. After excluding duplicates, the titles and abstracts of 1303 studies were screened. A total of 1260 studies were excluded since they were either duplicates or irrelevant to the topic. Thereafter, we completed a comprehensive assessment of the full text of 43 articles. A total of 39 articles were deemed ineligible during the full-text assessment, with the specific reasons for exclusion detailed in Fig. 1. Finally, our meta-analysis included 4 RCTs.

### 3.2 Characteristics of Included Studies

Table 1 (Ref. [13, 21, 22, 23]) provides the characteristics of the included studies. The included studies were all RCTs, which included 1096 participants [13, 21, 22, 23]. The average age of patients in all trials was over 60 years old. Two trials were performed in the United States [13, 23], while the other two studies were conducted in Europe [21, 22]. Three studies only reported POD [13, 22, 23], whereas only one reported POD and POCD [21]. Three of the studies mentioned the postoperative follow-up time [22].

**Table 1.** **Characteristics of included randomized controlled trials**.

Author	Country	Population	Intervention	POD/POCD assessment method	Follow-up period
Wildes *et al*. 2019 [13]	United States	A total of 459 patients with a mean age older than 60 years	EEG: BIS = 230	CAM and CAM-ICU	POD (postoperative days 1–5)
			Control: usual care = 229		
Kunst *et al*. 2020 [21]	United Kingdom	A total of 82 patients with a mean age older than 70 years	EEG: BIS = 42	CAM and MMSE	POD (postoperative day 3–5)
			Control: usual care = 40		POCD (postoperative day 3–5, 6 weeks and 1 year)
Whitlock *et al*. 2014 [23]	United States	A total of 310 patients with a mean age older than 60 years	EEG: BIS = 149	CAM-ICU	POD (postoperative day 1–10 or ICU discharge)
			Control: ETAC = 161		
Sponholz *et al*. 2020 [22]	Germany	A total of 245 patients with a mean age older than 65 years	EEG: visible-NT = 122	CAM-ICU	Not reported
			Control: blinded-NT = 123		

Abbreviations: EEG, electroencephalography; ETAC, end-tidal anesthetic concentration; CAM, Confusion Assessment Method; CAM-ICU, Confusion Assessment Method for The Intensive Care Unit; MMSE, Mini-Mental State Examination; BIS, bispectral index; POD, postoperative delirium; POCD, postoperative cognitive dysfunction; NT, Narcotrend.

### 3.3 Risk of Bias in Included Studies

**Fig. 2.**
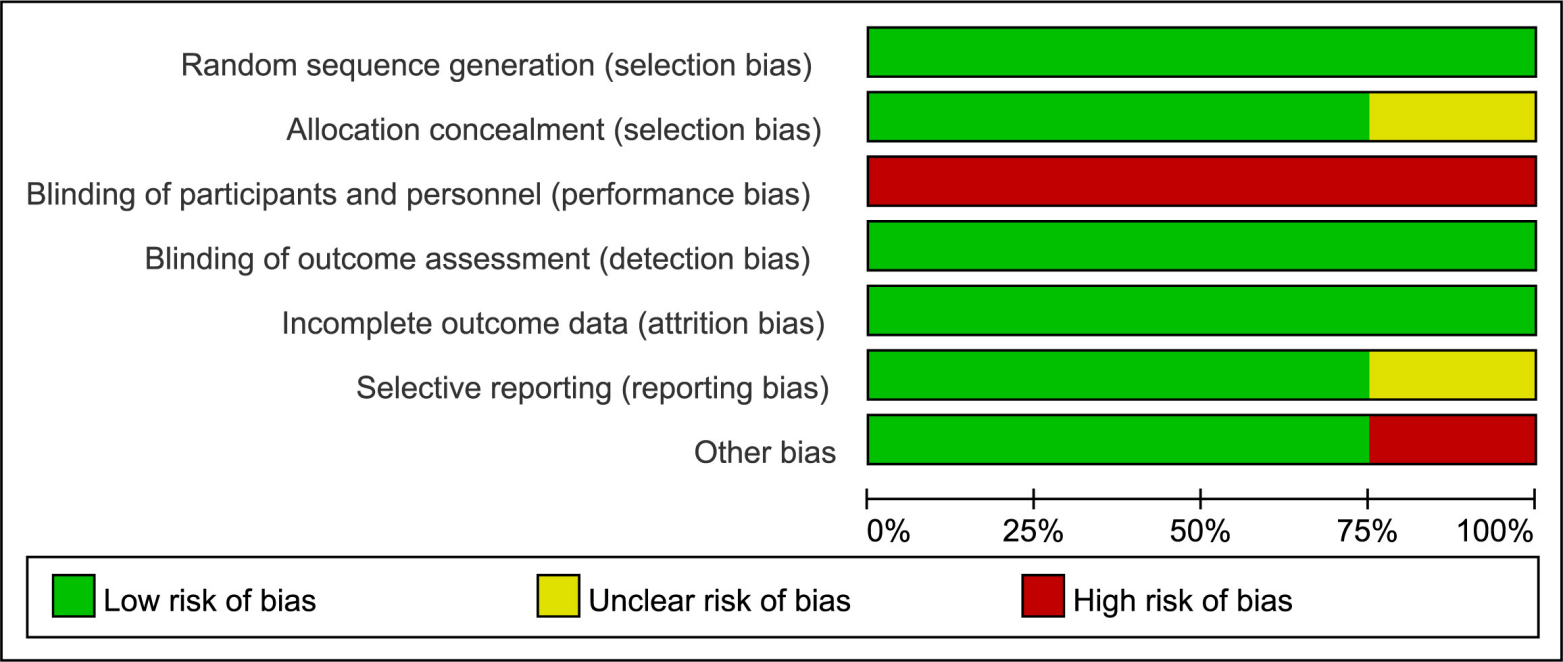
**Risk of bias graph for all included studies**.

**Fig. 3.**
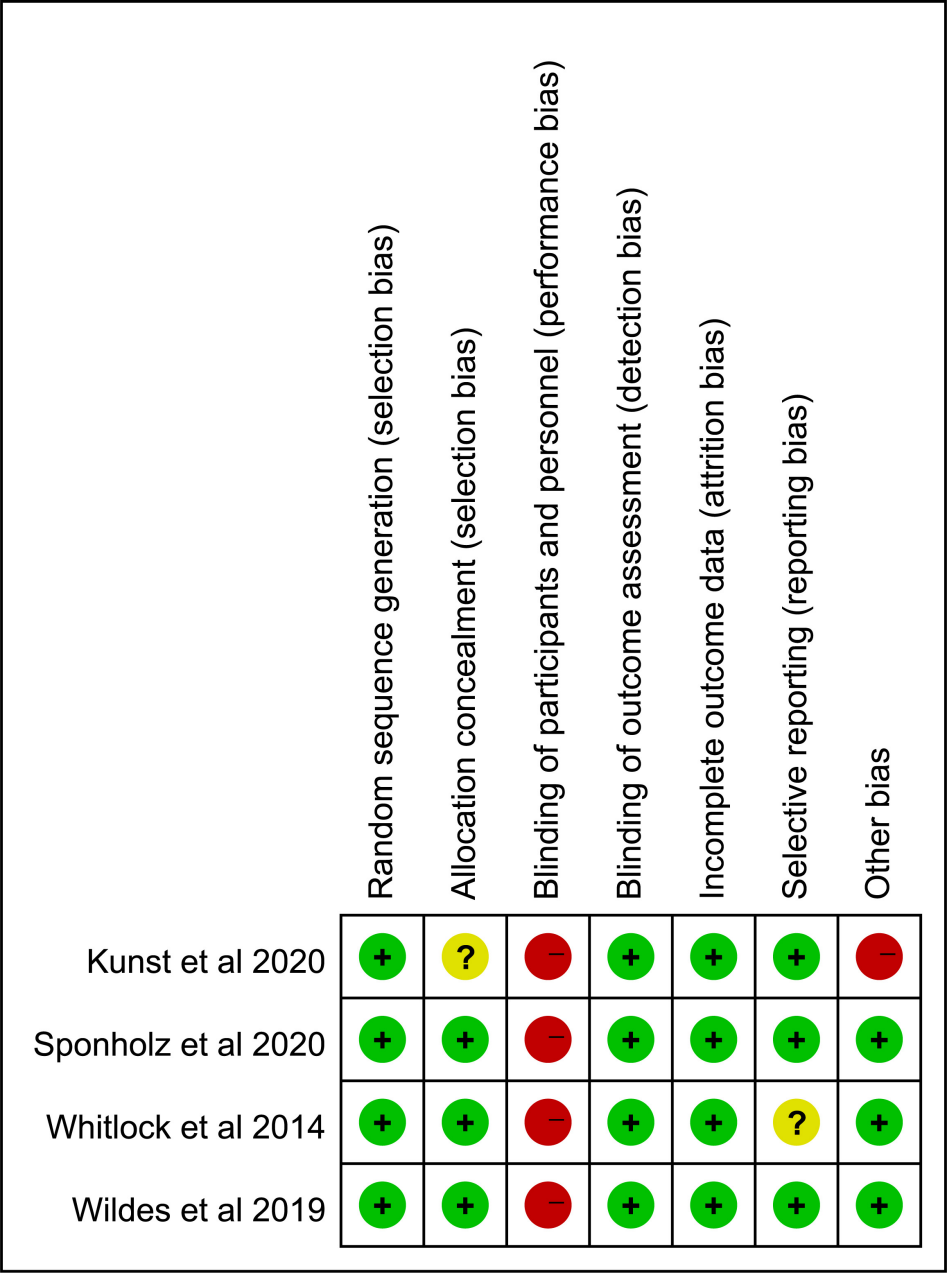
**Risk of bias for each included study**.

Figs. 2,3 summarize the risk of bias in each study. All included trials reported the generation of random sequences. There was one trial that did not disclose their method of allocation concealment [21]. It was impossible to blind the anesthesiologists to the electroencephalogram group. Therefore, all trials showed high risks of performance bias. Blinding the outcome assessment was mentioned in all studies. The absence of trial registration information in one study made it uncertain whether it was at risk of selective reporting [23].

### 3.4 Primary Outcomes

**Fig. 4.**
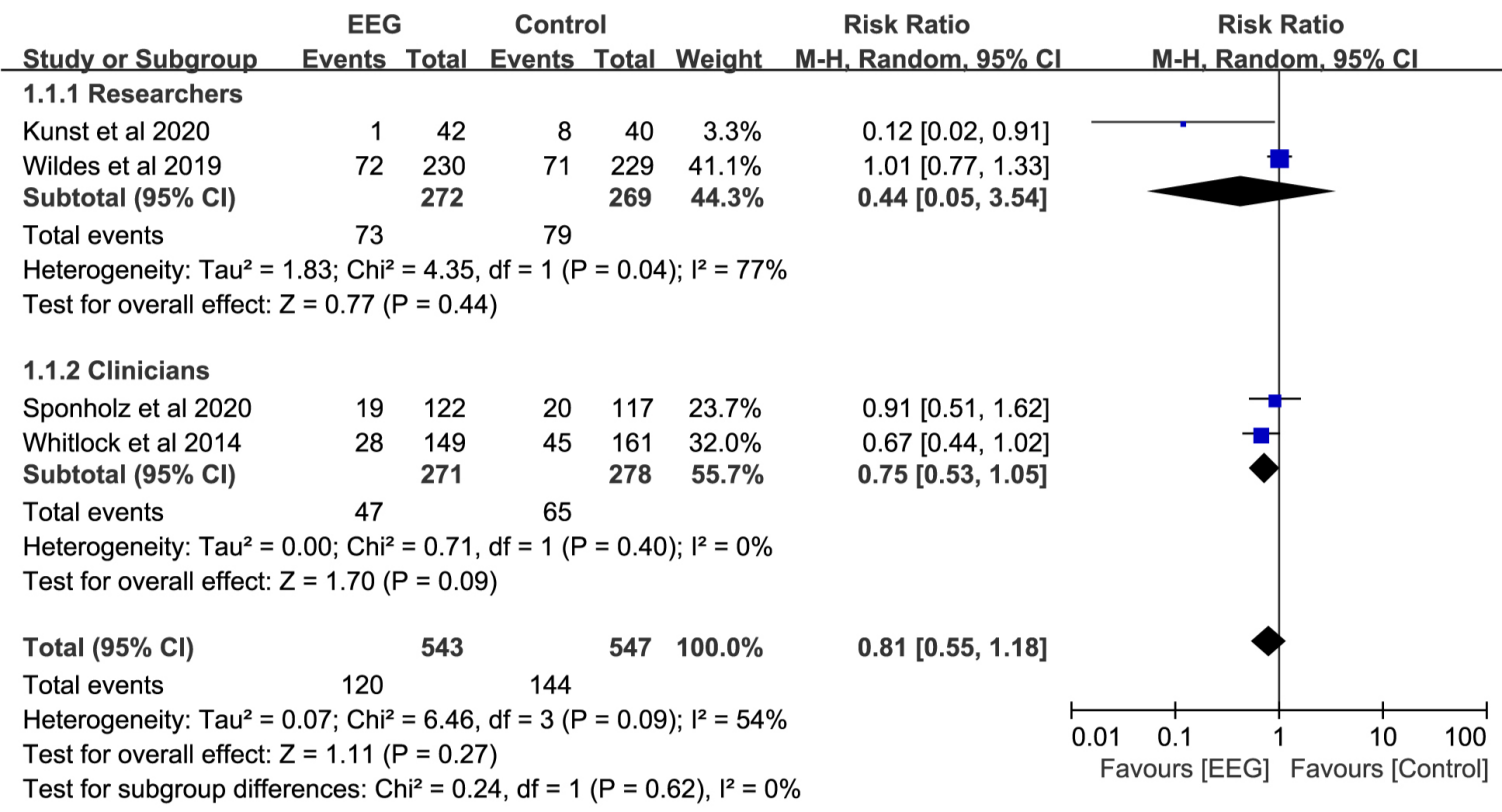
**Forest plots of postoperative delirium for the EEG group vs. control group**. EEG, electroencephalography; M–H, Mantel–Haenszel; CI, confidence interval.

The primary outcomes of this meta-analysis included POD and POCD, with the incidence of POD reported in all four studies. Due to the high heterogeneity (*$I^{2}$* = 54%), we used a random effects model to combine and analyze the data. Our meta-analysis, described in Fig. 4, found no significant correlation between EEG monitoring and lower POD risk (RR: 0.81; 95% CI: 0.55–1.18; *p* = 0.270). Assessors are a significant potential confounder affecting the accuracy of delirium evaluations [24]. We conducted a subgroup analysis based on the type of evaluator. The results indicated that EEG monitoring was unable to reduce the incidence of POD in either the clinician subgroup (RR: 0.75; 95% CI: 0.53–1.05; *p* = 0.090) or the researcher subgroup (RR: 0.44; 95% CI: 0.05–3.54; *p* = 0.440) (Fig. 4).

**Fig. 5.**
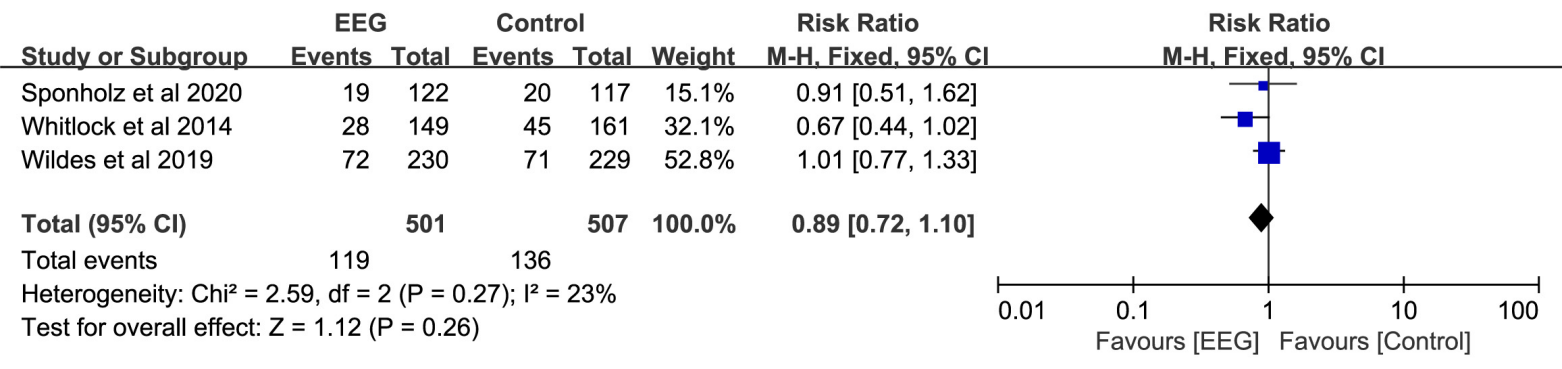
**Sensitivity analysis for postoperative delirium for the EEG group vs. control group**. EEG, electroencephalography; M–H, Mantel–Haenszel; CI, confidence interval.

We conducted a sensitivity analysis of the included literature to identify any outlier trials that may be responsible for the observed differences. The removal of the trial by Kunst *et al*. [21] resulted in the highest reduction in heterogeneity (*$I^{2}$* = 23%), and the sensitivity analysis result was consistent with the original outcome (RR: 0.89; 95% CI: 0.72–1.10; *p* = 0.260) (Fig. 5). Overall, our findings were noticeably consistent.

A meta-analysis of the POCD risk was not feasible due to limited data. Only the study by Kunst *et al*. [21] reported the incidence of POCD. They found no significant differences in POCD risk between the EEG and control groups at any follow-up point.

### 3.5 Secondary Outcomes

**Fig. 6.**
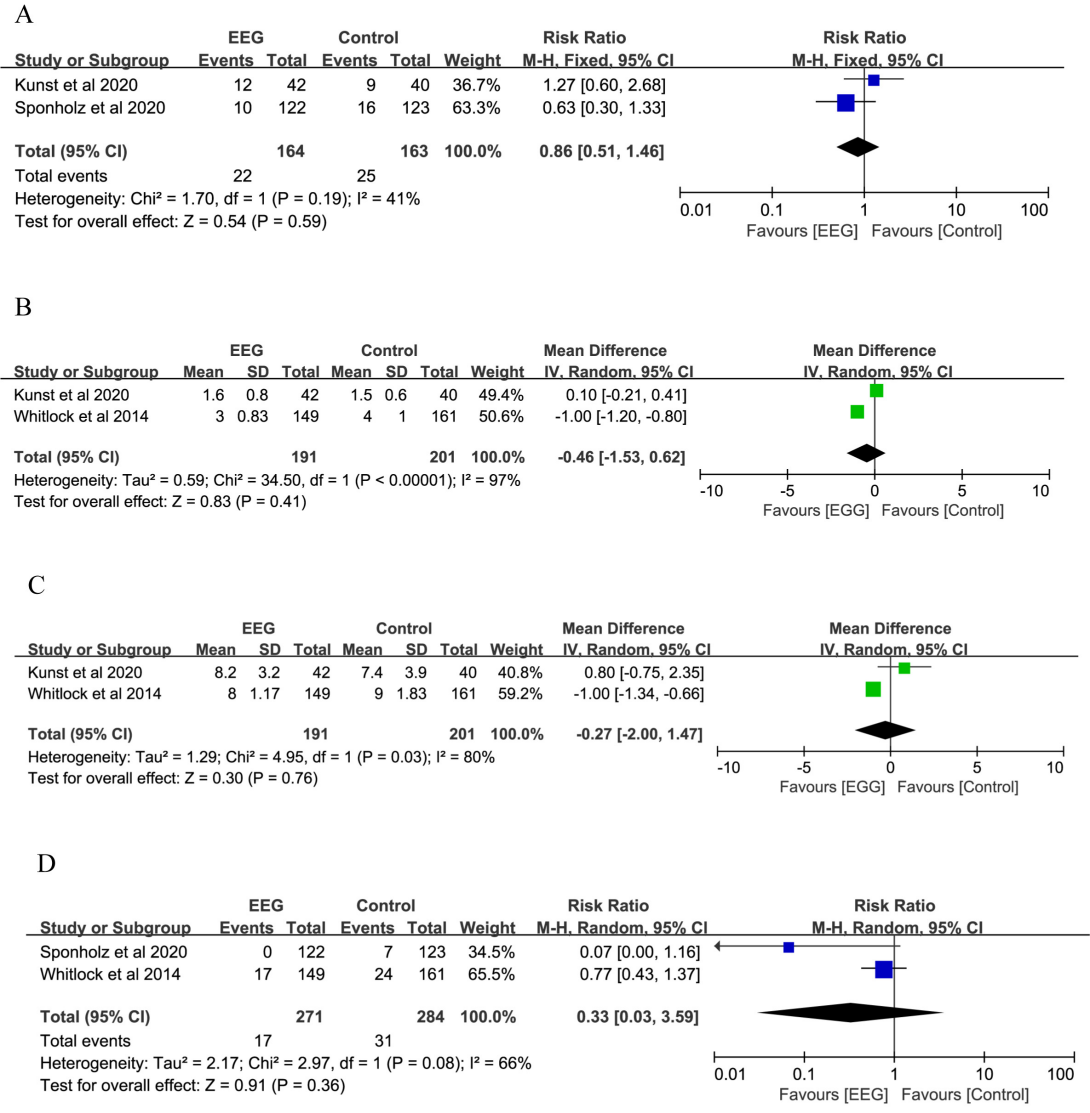
**Forest plots of secondary outcomes for the EEG group vs. control group**. (A) Forest plots of red cell transfusions; (B) forest plots of the length of ICU stay (days). (C) Forest plots of the length of hospitalization (days). (D) Forest plots of mortality. EEG, electroencephalography; M–H, Mantel–Haenszel; IV, inverse variance; CI, confidence interval; ICU, intensive care unit.

We analyzed other clinical outcomes, including red cell transfusion, intensive care unit (ICU) stay, hospital stay, mortality, myocardial and cerebral injury markers, and adverse events. The results of the two studies showed that EEG monitoring did not effectively reduce the need for red cell transfusions (RR: 0.86; 95% CI: 0.51–1.46; *p* = 0.590) (Fig. 6A). EEG monitoring also did not significantly reduce the length of the ICU stay (MD: –0.46; 95% CI: –1.53–0.62; *p* = 0.410), hospital stay (MD: –0.27; 95% CI: –2.00–1.47; *p* = 0.760), and mortality (RR: 0.33; 95% CI: 0.03–3.59; *p* = 0.360) (Fig. 6B–D).

Insufficient data prevented analyses of myocardial injury, cerebral functions, and other adverse events. However, Kunst *et al*. [21] found no significant differences in myocardial and cerebral injury biomarkers between the intervention group and the control group, including troponin I, matrix metalloproteinase 9 (MMP9), ubiquitin carboxy-terminal hydrolase L1 (UCHL1), and glial fibrillary acidic protein (GFAP). In terms of adverse events, Kunst *et al*. [21] reported similar rates of new-onset atrial fibrillation, infection, and acute kidney injury in both groups. Sponholz *et al*. [22] found that the visible-Narcotrend (NT) group had a lower incidence of intraoperative adverse events than the blinded-NT group (*p* = 0.010).

## 4. Discussion

This meta-analysis comprised 4 RCTs with 1096 participants and evaluated the effect of EEG monitoring on POD and POCD in cardiothoracic surgery patients. Our findings showed that EEG monitoring did not result in a significant reduction in the incidence of POD. However, a meta-analysis could not be performed for POCD due to insufficient data. Notably, Kunst *et al*. [21] reported no significant differences in POCD risk between the intervention and control groups at any follow-up point. No statistical differences in red cell transfusions, length of ICU and hospital stays, and mortality between the EEG and control groups were also observed in our pooled results.

It has been suggested that EEG could be a potential tool for reducing POD occurrence [25]. The use of EEG during surgery is effective in decreasing the duration of burst suppression and minimizing exposure to anesthesia [26]. Evidence has suggested that burst suppression was closely connected to the risk of POD and POCD [27, 28]. Fritz *et al*. [29] found that patients who experienced prolonged burst suppression during surgery were more likely to develop POD. Previous clinical studies supported the benefits of EEG monitoring in decreasing the risks of both POD and POCD [11, 30, 31]. Chan *et al*. [11] conducted an RCT with 902 patients and found that bispectral index (BIS)-guided anesthesia reduced the risk of POD and POCD. Another prospective controlled trial involving 81 patients also showed that the BIS group had a lower incidence rate of POD than the non-BIS group [30]. In addition, Bocskai *et al*. [31] conducted a meta-analysis of 14 RCTs to investigate the protective effect of EEG monitoring. Their meta-analysis results suggested that EEG could reduce the incidence of POD and POCD. Based on our meta-analysis, it was observed that EEG monitoring did not provide any protection against POD in patients undergoing cardiothoracic surgery. For the incidence of POCD, Kunst and his colleagues found that EEG monitoring was ineffective in preventing POCD six weeks after surgery [21]. To exclude the impact of high heterogeneity between the included literature and the results, we performed a sensitivity analysis by excluding one study at a time. The heterogeneity in the POD meta-analysis was significantly reduced following the removal of the study by Kunst and colleagues [21]; however, the primary results did not show any significant change, indicating that our meta-analysis results were relatively stable.

Multiple pathogenic mechanisms contribute to the occurrence of POD and POCD, including neuroinflammation [32], neurotransmitter disorders [33], and intestinal homeostasis disorder [34]. Particularly, the mechanisms are more complex and diverse in the POD and POCD development process after cardiothoracic surgery. Compared to other general surgeries, cardiothoracic surgery is considered to have a higher risk of POD [35]. In cardiothoracic surgery, surgical stress leads to systemic inflammation. Cardiopulmonary bypass (CPB) also may worsen neuroinflammation and cause microembolization in the brain [17, 36]. Glumac *et al*. [37] discovered that administering corticosteroids before surgery reduced the inflammatory response, thereby decreasing the incidence and severity of POCD. Sun *et al*. [38] conducted a meta-analysis of five RCTs to examine the connection between POD risk and EEG monitoring. The study discovered that EEG monitoring did not prevent POD. Furthermore, they believed that the results of the analysis could be impacted by the inclusion of two trials with cardiac surgery patients. Another meta-analysis, which investigated the impact of EEG on PND, showed that EEG monitoring was associated with a lower PND incidence rate [19]. However, their subgroup analysis of patients undergoing cardiothoracic surgery showed that EEG monitoring did not lower the risk of PND. Our results further demonstrated that EEG monitoring had a limited effect on preventing POD in patients undergoing cardiac surgery. Comprehensive management can be considered to help prevent POD and POCD, given their multiple pathogenesis.

This is the first meta-analysis to investigate the effect of EEG monitoring on POD and POCD in patients undergoing cardiothoracic surgery. Our analysis results are valuable in the development of clinical guidelines for cardiothoracic surgery. Certain variances in the included studies could potentially explain our results. Previous evidence has suggested that maintaining appropriate anesthesia depth with EEG monitoring could reduce the incidence of POD [39]. However, statistical differences in the anesthesia depth were not observed in all EEG groups in the studies compared to the control group. Additionally, the number of patients undergoing deep anesthesia did not differ between the EEG and control groups in the RCT conducted by Whitlock and colleagues [23]. The tools utilized for POD evaluation are also somewhat different. Confusion Assessment Method for The Intensive Care Unit (CAM-ICU) instruments were used to measure POD in two studies with limited sensitivity in non-intubated patients [40]. In addition, the patients analyzed in the two RCTs have a high average age and American Society of Anesthesiologists (ASA) grade, which are considered high-risk factors for preoperative procedures [13, 23].

Consideration of certain limitations is required when evaluating this meta-analysis. (1) Due to the limited number of studies referenced in this meta-analysis, we could not conduct a publication bias analysis. (2) The universality of the results may be limited by the average age of patients included in this study, which was above 60 years. (3) The study by Wildes *et al*. [13] contributed 42% of the patients included in this meta-analysis, meaning the results from their study had a significant impact on the overall effect of this study. (4) The studies included in this meta-analysis revealed different evaluation tools, frequencies, and periods that could impact the detection of POD. (5) Only one RCT reported on the POCD risk in this meta-analysis. Kunst *et al*. [21] performed the Mini-Mental State Examination (MMSE) to assess the postoperative cognitive function of the patients on postoperative days 3–5, at 6 weeks, and one year. However, they did not clearly define either POCD or delayed neurocognitive recovery in the study. In the current literature, there is a lack of consistency in the methods for assessing POCD and delayed neurocognitive recovery. MMSE also has capping and learning effects and is not sensitive to subtle cognitive changes that may occur after surgery. Therefore, our meta-analysis is insufficient to explore the preventive effect of EEG monitoring on POCD risk. Furthermore, each secondary outcome analysis consisted of two or three RCTs, and the studies had a high degree of heterogeneity. As a result, these findings require further investigation to confirm their accuracy.

## 5. Conclusions

This meta-analysis did not find evidence supporting EEG monitoring as a potential method to reduce the incidence of POD in patients with cardiothoracic surgery. In the future, more high-quality RCTs with large sample sizes are needed to validate the relationship between EEG monitoring and POD/POCD further.

## Data Availability

All data and materials were from published research.

## Supplementary Material

Supplementary material associated with this article can be found, in the online version, at https://doi.org/10.31083/j.rcm2504126.
